# Thermo-Responsive Hydrogel-Based Soft Valves with Annular Actuation Calibration and Circumferential Gripping

**DOI:** 10.3390/bioengineering8090127

**Published:** 2021-09-20

**Authors:** Manivannan Sivaperuman Kalairaj, Hritwick Banerjee, Kirthika Senthil Kumar, Keith Gerard Lopez, Hongliang Ren

**Affiliations:** 1 Department of Biomedical Engineering, National University of Singapore, Singapore 117575, Singapore; biesikm@nus.edu.sg (M.S.K.); biehb@nus.edu.sg (H.B.); s.kirthika@u.nus.edu (K.S.K.); keithglopez@u.nus.edu (K.G.L.); 2 Department of Electronic Engineering, The Chinese University of Hong Kong, Central Ave., Hong Kong, China

**Keywords:** hydrogels, thermo-responsive polymers, valves, soft actuators, soft gripper

## Abstract

Valves are largely useful for treatment assistance devices, e.g., supporting fluid circulation movement in the human body. However, the valves presently used in biomedical applications still use materials that are rigid, non-compliant, and hard to integrate with human tissues. Here, we propose biologically-inspired, stimuli-responsive valves and evaluate N-Isopropylacrylamide hydrogels-based valve (NPHV) and PAAm-alginate hydrogels-based valve (PAHV) performances with different chemical syntheses for optimizing better valve action. Once heated at 40 ${}^{\circ }$C, the NPHV outperforms the PAHV in annular actuation (NPHV: 1.93 mm displacement in 4 min; PAHV: 0.8 mm displacement in 30 min). In contrast, the PAHV exhibits a flow rate change of up to 20%, and a payload of 100% when the object is at 100 ${}^{\circ }$C. The PAHV demonstrated a completely soft, stretchable circular gripper with a high load-to-weight ratio for diversified applications. These valves are fabricated with a simple one-pot method that, once further optimized, can offer transdisciplinary applications.

## 1. Introduction

In the human body, biological control regulates complex mechanisms ranging from the microscopic (e.g., cellular mechanotransduction, membrane potential) to the macroscopic (e.g., thermoregulation). For example, a sudden drop in the ambient temperature leads to blood vessel contraction and yields to shivering [1]. These biochemical processes lead to inspiration for engineering materials to mimic the stimuli-responsive mechanism as an efficient energy conversion mechanism (e.g., chemical to mechanical). In this aspect, stimuli-responsive hydrogels can play a pivotal role [2]. Hydrogel-based, stimuli-responsive polymers in recent years have become an integral part of biocompatible, soft biomedical actuators and sensors [3,4,5,6,7,8]. Unlike conventional actuators, stimuli-responsive hydrogels can control volume change without the necessity of any external power source [9,10,11,12,13]. For example, poly(N-isopropylacrylamide) (NIPAAm) transforms coil-to-globule transition, which further leads to lower critical solution temperature (LCST) [14]. A detailed rheological analysis can be useful in choosing thermoresponsive hydrogels and their pseudo-plasticity with the viscoelastic transition [15,16]. This has a significant impact that in turn offers a lightweight and biocompatible system with fine control [9,17,18,19,20,21]. Application avenues of these mechanically-compliant biomaterials include soft robotics [22], stretchable electronics [23], artificial skin [24], safer human–machine interaction [25], biologically-inspired valves [26], drug release [27], microfluidics [28], and more [29,30,31].

Inside the field of biologically-inspired valves, pH, temperature, and external mechanical stimuli have demonstrated viable actuation performance [32,33,34]. Even though decades of studies have been performed regarding hydrogel-based organ replacement and downstream physiological interactions, the field of valve replacement is still in its nascent phase [35,36,37]. The actuation scenario for designing a viable hydrogel-based valve is still concerned with slow response, and energy efficiency is at stake. NIPAAm, an excellent thermo-responsive smart polymer, has been presented as a nano-valve with inverse solubility upon heating [38]. It has also been shown that the addition of polydopamine enhances the degree of immobilization, leading to controlled membrane protein adsorption [39]. In addition, NIPAAm was also demonstrated as an excellent micro-valve for controlled drug delivery systems [40,41,42]; nevertheless, little to no literature support a mesoscale valve to date. Recently, polyacrylamide(PAAm)-alginate-based hydrogels, due to their swelling/de-swelling, low modulus nature [43,44], have also been introduced as the soft matrix for fabricating a tricuspid valve [45]. The PAAm-alginate-based hydrogels are, however, only transformed by volume and not by controlled shape changes. One approach to improve shape morphing would be to introduce multiple active layers with different stimuli-responsive natures for twisting [46,47,48], buckling [49,50], and bending [46,51,52]. Even with these initial promises, synthetic hydrogels are no comparison to heart valves with their extraordinary fatigue-resistant properties. It is indeed necessary to scale up the process and fabricate macroscopic biocompatible valves that will be fatigue-resistant for implanting in vivo.

From a cytotoxicity/tissue engineering standpoint, there are several demonstrations of alginate gels used as potentially promising biomaterials for 3D printed heart valve conduits that anatomically resemble in vivo counterparts [53,54]. However, 3D bioprinting and regenerative medicine-driven organ transplantation are still far from their true potential. In this spirit, here we demonstrate a N-Isopropylacrylamide hydrogels-based valve (NPHV) that provides a new outlook based on temperature changes, and compares valve performance with previously established PAAm-Alginate hydrogels [8]. During tissue ablation and focused ultrasound treatment, the localized elevated tissue temperature can expedite the valve action and deploy it in the targeted position. In our initial pilot [10] study, we compared NPHV performance embedded with shape memory alloys (SMAs)-based actuation. SMAs, in general, are highly effective, resilient, possess high energy density, and are scalable for faster actuation and hence applied in soft, compliant robotics applications [55,56]. Here, we follow thermally-responsive hydrogel synthesis to use all soft, compliant valves for potential bioengineering applications. From a soft robotics perspective, we deploy an initial proof-of-concept gripper for maximum payload.

## 2. Materials and Methods

### 2.1. Materials

Acetone (Sigma-Aldrich; ≥99.5%), ethanol (Sigma-Aldrich; ≥99.5%), methanol (Sigma-Aldrich; ≥99.9%) remained employed as received without additional purification. For hydrogel synthesis, acrylamide (AAm, Sigma-Aldrich; ≥99.9%), N-Isopropylacrylamide (NIPAAm, Sigma-Aldrich; ≥99%), sodium alginate (SA, Sigma-Aldrich; ≤15.5%), 2-hydroxy-4${}^{\prime }$-(2-hydroxyethoxy)-2-methylpropiophenone (photo-initiator or PI, Sigma-Aldrich; ≥97.5%), N, N${}^{\prime }$-Methylenebis(acrylamide) (MBAA, Sigma-Aldrich; ≈99%), ammonium persulfate (APS, Sigma-Aldrich; ≈98%), N,N,N${}^{\prime }$,N${}^{\prime }$-Tetramethylethylendiamin (TEMED, Sigma-Aldrich; ≈99%), and deionized (DI) water (Milli-Q pore; 18.2 MΩ) were used as received without additional modification. Poly(dimethylsiloxane) (PDMS) (Sylgard 184, Dow Corning) was prepared as prescribed with the 10:1 base-to-catalyst ratio. The adhesive (Scotch) tape was purchased from 3M.

### 2.2. NPHV Manufacturing

**Figure 1:**
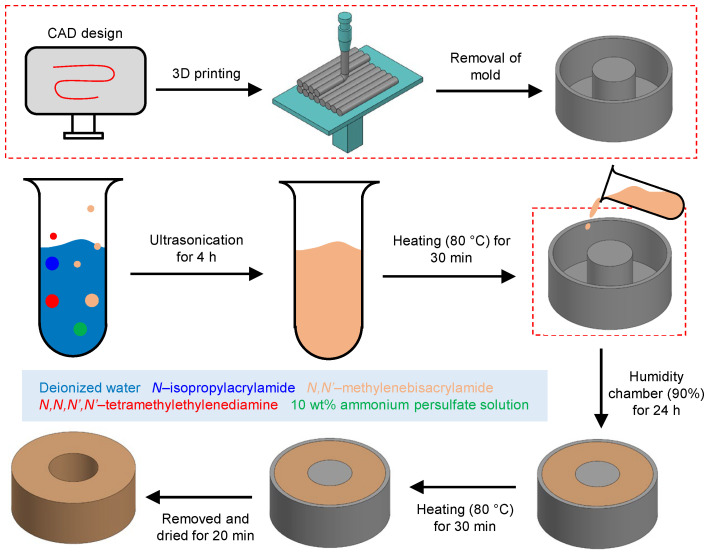
Steps involved in the fabrication of NPHV.

NPHVs were fabricated by the soft-casting method with the thermally-responsive hydrogel solution. The steps involved in the fabrication of NPHVs are portrayed in Figure 1. An amount of 1.2 g of NIPAAm, a thermo-responsive monomer that induces the hydrogel valve to respond to temperature changes, was mixed with 20 mg of MBAA in a 50 mL vial and dissolved with 6 ml of DI water [57]. MBAA acted as a cross-linker, creating a branched polymer hydrogel. Then, 200 μL of 10 wt.% APS solution and 120 μL of TEMED were introduced into the vial as an initiator and accelerator, respectively, for the polymer formation process.

The thermo-responsive hydrogel was then immediately poured into the PDMS molds and placed in a humidified chamber (humidity ≈ 90%) for 24 h for polymerization. PDMS molds were fabricated by the soft cast method using a 3D printer (LulzBot, Aleph Objects). The polymerized hydrogel was then carefully extracted from the PDMS molds and immersed in DI water at 20 ${}^{\circ }$C for 2 h. Hydrogels were then placed on a PDMS support with a 3 mm-diameter needle inserted through the lumen and heated in an oven (80 ${}^{\circ }$C) for 30 min. The hydrogel shrank and returned to its basal dry state while the needle maintained its overall shape. The freshly synthesized hydrogel was then coated with an adhesive (following [57]), then dried for 20 min. As previously discussed, hydrogels with adhesive demonstrated high gripping strength due to both the adhesiveness of the surface and the bulk compressive force derived from the hydrogel’s directed expansion [57,58]. These procedures were followed with removal of the needle and immersion of the hydrogel in water for 20 min, after which the hydrogel was ready to use.

### 2.3. PAHV Manufacturing

**Figure 2:**
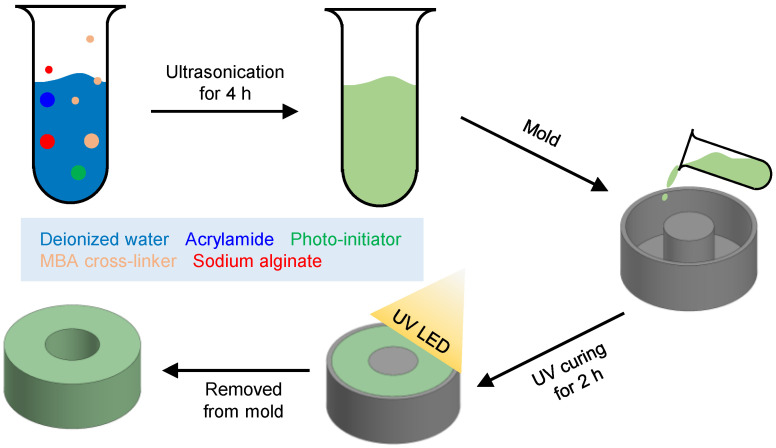
Steps involved in the fabrication of PAHV.

PAHVs were manufactured by the soft-casting method with the PAAm-alginate hydrogel solution. Detailed steps involved are overlaid in Figure 2. The adapted one-pot method [8,19,59] was used to prepare stock solutions comprised of AAm, MBAA, PI, SA, and DI water. The resultant stock solution kept in the ultrasonic cleaner (Sonica 5200, Sonica) with continuous stirring for 4 h followed by a 24 h rest period for crosslinking. The percentage of the weight used for synthesizing PAAm-alginate hydrogel was AAm of 22.6 %wt., SA of 1.5 %wt., PI of 0.58 %wt., MBAA of 0.020 %wt., DI water of 75.3 %wt., as depicted without modification from [60].

Freshly prepared PAAm-alginate hydrogel mix was poured into the PDMS molds and placed in a humidity chamber (humidity ≈ 90%) for 24 h for polymerization. The same dimension of PDMS molds was used as with the NPHVs. The polymerized hydrogel was then carefully extracted from the PDMS molds and immersed in DI water at 20 ${}^{\circ }$C for 2 h. The PAAm-alginate hydrogel was then placed on a PDMS support with a 3 mm-diameter needle inserted through the lumen and heated in an oven (80 ${}^{\circ }$C) for 30 min. The PAAm-alginate hydrogel shrank and returned to its basal dry state while the needle maintained its overall shape. The hydrogel matrix was then coated with an adhesive and dried for 20 min, followed by removal of the needle and immersion of the hydrogel in water for 20 min, after which the hydrogel was ready to use.

### 2.4. Actuation Mechanism

NPHV actuation was performed by immersing samples in 100 mL of DI water in a glass beaker and heating it using a hot plate (SP88857200, Fisher Scientific, Hampton, NH, USA). Exposure of the NPHVs to heat activates the thermo-responsive nature of the prototype and induces expansion of the lumen while maintaining its outer diameter, demonstrating a valve-like mechanism. Before actuating, a video camera was placed normal to the actuation plane of the NPHV sample and the entire actuation process was recorded. The trajectory of the lumen expansion in the video was then analyzed using “Tracker” (https://physlets.org/tracker/, accessed on 12 September 2020) to obtain the time-resolved displacement of the NPHV. PAHV actuation, however, was performed by placing the PAHV samples on a hot plate to initiate swelling/de-swelling mechanics. PAHV samples were monolithically integrated and, like NPHV, temporal deformation was analyzed. During PAHV actuation, thermal profiles were captured using infrared (IR) imaging (FLIR One Pro, FLIR Systems, Wilsonville, OR, USA) by placing the IR camera normal to the actuation plane at a distance of 50 mm.

### 2.5. Dehydration/Rehydration Kinetics

PAHV weight loss during actuation was determined using a precision balance (EP 125SM, Precisa Gravimetrics). PAHV samples’ initial weights (w${}_{0}$) were measured and recorded before actuation. During actuation, the PAHV samples’ weights (w) were measured every 5 min. PAHV samples’ weight ratios at each instant were calculated using w/w${}_{0}$. During the rehydration, the weights (w) of each PAHV sample were measured and the weight ratios (w/w${}_{0}$) were calculated.

### 2.6. Finite Element (FE) Simulations

The FE simulations of the heat transfer in the PAHV during the actuation process were carried out using the heat transfer module of COMSOL Multiphysics 5.3 using a time-dependent equation, following the data from our previous work [8]:

(1) $$d_{z}\rho C_{p}\frac{\partial T}{\partial t}+d_{z}\rho C_{p}u▿T+▿q=d_{z}Q+q_{0}+d_{z}Q_{ted}$$

where $\frac{\partial T}{\partial t}$ is the temperature gradient, ρ is the density of the PAHV measured in kg/m${}^{3}$, $d_{z}$ is the thickness of the cross-section measured in mm, ▿T is the temperature change in the PAHV measured in K, *Q* is the heat energy measured in kJ, the specific heat capacity at constant pressure ($C_{p}$) is estimated to be ≈2.5 kJ/kg K [61], and *q* is given as

(2) $$q=-d_{z}k▿T$$

where the thermal conductivity of the PAHV (*k*) is ≈0.5 W/kg K [62]. A temperature of 100 ${}^{\circ }$C was applied to the bottom layer of the PAHV cross-section which was at 20 ${}^{\circ }$C and the temperature change of the cross-section was monitored until the entire cross-section reached 100 ${}^{\circ }$C. During the analysis, the effect of dehydration and volume change in the PAHV cross-section was not considered.

## 3. Results

### 3.1. NPHV’s Thermo-Mechanical Performance

**Figure 3:**
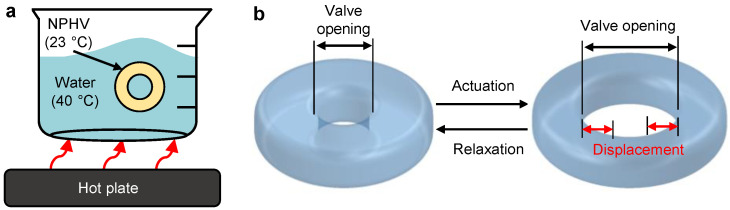
Actuation of the NPHV. (**a**) Schematic showing the experimental set-up of the NPHV actuation. (**b**) Schematic demonstrating NPHV’s actuation mechanism.

**Figure 4:**
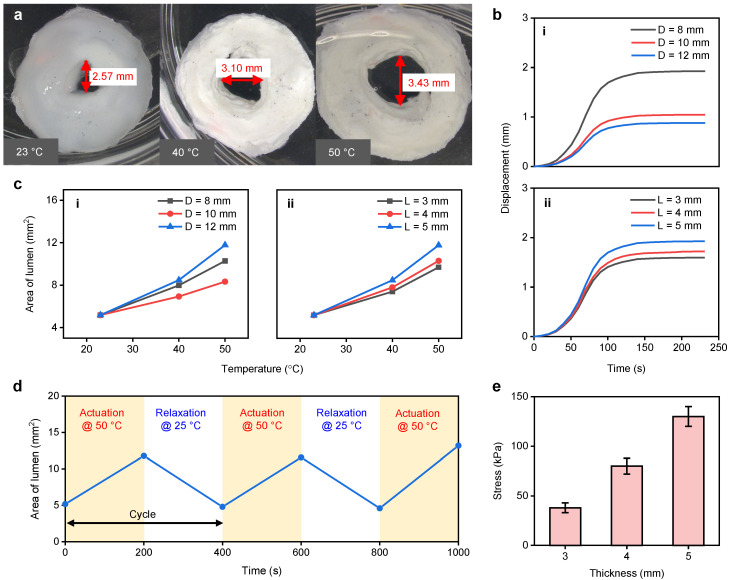
Thermo-mechanical performance of NPHVs. (**a**) Optical microscopy images showing the displacement of NPHV when actuated at 40 and 50 ${}^{\circ }$C. (**b**) (i) Displacement in NPHVs (*L* = 5 mm) with different diameters (*D* = 8–12 mm) when actuated at 40 ${}^{\circ }$C for 230 s. (ii) Displacement in NPHVs (*D* = 8 mm) with different thicknesses (*L* = 3–5 mm) when actuated at 40 ${}^{\circ }$C for 230 s. (**c**) (i) Changes in NPHV lumen area (*L* = 5 mm) for different diameters (*D* = 8–12 mm) when actuated at 40 and 50 ${}^{\circ }$C. (ii) Changes in NPHV lumen area (*D* = 12 mm) for different thicknesses (*L* = 3–5 mm) when actuated at 40 and 50 ${}^{\circ }$C. (**d**) NPHVs’ lumen area change (*D* = 12 mm, *L* = 5 mm) over three cycles actuated at 50 ${}^{\circ }$C. (**e**) NPHVs’ compressive strength at different thicknesses (*L* = 3, 4, and 5 mm).

We designed five different variations of NPHV, with all designs having the same lumen diameter (*d* = 4 mm) but different outer diameters (*D* = 8, 10, and 12 mm) and heights (*L* = 3, 4, and 5 mm). For performance testing, NPHVs were immersed in a water bath for 230 s, maintained at 40 ${}^{\circ }$C by a hot plate (SP88857200, Fisher Scientific, Hampton, NH, USA), as shown in Figure 3a. Since the lower critical solution temperature (LSCT) of NPHV is ≈30–35 ${}^{\circ }$C[63], increasing the temperature above LCST induces shrinking, resulting in a valve-like mechanism. NPHVs also demonstrated large variations in nominal stresses between 31 ${}^{\circ }$C (5 kPa) and 39 ${}^{\circ }$C (20 kPa) [64]. Due to the requirement of higher stresses for the application, the TRHVs were tested at 40 ${}^{\circ }$C. Heat exposure activates the thermo-responsive nature of the NPHV prototype and induces lumen expansion while maintaining its outer diameter, demonstrating a valve-like mechanism, as shown in Figure 3b. The NPHV actuation process was then video-recorded, and the lumen trajectory expansion was analyzed using “Tracker” (https://physlets.org/tracker/, accessed on 12 September 2020) to obtain the NPHV’s time-resolved displacement over 230 s. Figure 4b shows the NPHVs’ five different displacement variations when heated at 40 ${}^{\circ }$C for 230 s, and all the variations demonstrated rapid displacement during the first 100 s of the actuation, followed by deceleration and stabilization at ≈210 s. A displacement of ≈0.88–1.93 mm was observed during the entire actuation period of 230 s, while the first 76 s demonstrated a displacement of ≈0.63–1.37 mm (71–72% of total displacement), enabling the possibilities of achieving more than 70% performance of the valve at thrice the frequency.

The NPHV with *D* = 8 mm shows the highest displacement (≈1.93 mm) while the NPHV with *D* = 12 mm shows the lowest displacement (≈0.88 mm), demonstrating that the lower the outer diameter, the higher the displacement during actuation. To evaluate the influence of the thickness of the NPHV in the actuation performance, NPHVs (*D* = 12 mm) with different thicknesses (*L* = 3, 4, and 5 mm) were actuated at 40 ${}^{\circ }$C for 230 s (Figure 4b(ii)). A displacement of ≈1.6–1.93 was observed in the NPHV with *D* = 8 mm for the thicknesses 3 mm to 5 mm. Figure 4b(ii) shows a similar trend in its displacement curve to that of Figure 4b(i), and shows that the change in thickness of the NPHV has a small influence on displacement performance.

All variations of NPHV were heated to 40 and 50 ${}^{\circ }$C and the lumens’ diameter changes were observed from optical microscopy (DMS1000, Leica Microsystems, Wetzlar, Germany) images. Figure 4a exhibits the optical microscopy images, demonstrating NPHVs’ lumen (*D* = 12 mm, *L* = 5 mm) diameter changes. Figure 4a displays an expansion of ≈0.21 times (3.1 mm) and 0.33 times (3.43 mm) the initial lumen size (2.57 mm) when heated at 40 ${}^{\circ }$C and 50 ${}^{\circ }$C, respectively. Figure 4c(i) demonstrates the NPHV lumens’ area change (*L* = 5 mm) with different outer diameters (*D* = 8–12 mm) actuated at 40 and 50 ${}^{\circ }$C for 230 s. The NPHVs with a lumen area of 5.18 mm${}^{2}$ at 23 ${}^{\circ }$C show an increase of ≈1.3–1.6 times (6.93–8.48 mm${}^{2}$) the lumen area when heated at 40 ${}^{\circ }$C, and an increase of ≈1.6–2.3 times (8.33–11.78 mm${}^{2}$) the lumen area when heated at 50 ${}^{\circ }$C. The NPHV with *D* = 12 mm shows the highest lumen area change at both 40 and 50 ${}^{\circ }$C while the NPHV with *D* = 10 mm shows the lowest. To evaluate the influence of NPHV thickness in actuation performance, NPHVs (*D* = 12 mm) with different thicknesses (*L* = 3, 4, and 5 mm) are actuated at 40 and 50 ${}^{\circ }$C for 230 s, and the corresponding changes in lumen area are determined (Figure 4b(ii)). The NPHV with *L* = 5 mm demonstrates the highest lumen area change at both 40 and 50 ${}^{\circ }$C while the NPHV with *L* = 3 mm exhibits the lowest, demonstrating that performance increase corresponds to the increase in thickness of NPHVs.

To examine the actuation reproducibility of the NPHVs, we actuated the NPHV (*D* = 12 mm, *L* = 5 mm) for three cycles with heating at 50 ${}^{\circ }$C for 200 s followed by cooling at room temperature (23 ${}^{\circ }$C) for 200 s, and changes in lumen area were observed (Figure 4d). Lumen area increased ≈2.3 times in 200 s during heating and reverted to its initial position during cooling (200 s). Lumen area changes in the subsequent actuation cycles were ≈2.4 times in the second cycle and ≈2.6 times in the third cycle. Different thicknesses of NPHV (*L* = 3, 4, and 5 mm) were tested for their compressive strength in a universal testing machine (5543, Instron) with a 1 kN load cell at a rate of 5 mm min${}^{-1}$. NPHVs showed a compressive strength of ≈38 kPa, ≈81 kPa, and ≈130 kPa for samples with thicknesses of 3, 4, and 5 mm, respectively (Figure 4e), demonstrating that the thicker the NPHV, the larger the compressive force exertion.

### 3.2. PAHV’s Thermal Actuation

**Figure 5:**
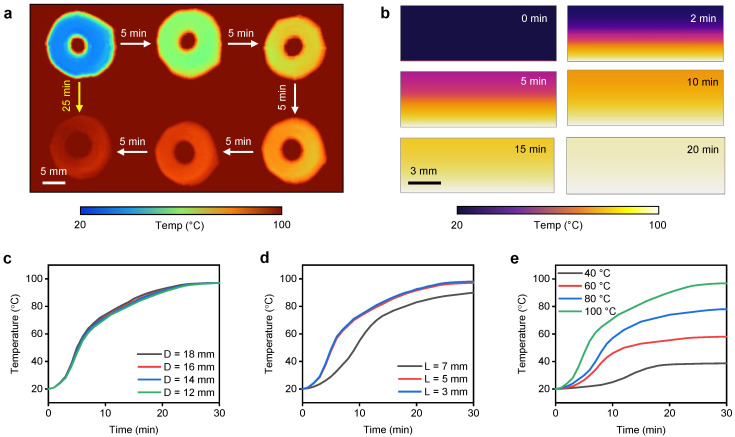
Thermal actuation of the PAHV. (**a**) IR images exhibiting the PAHV’s actuation at 100 ${}^{\circ }$C. (**b**) FE simulations showing heat transfer in the PAHV when actuated at 100 ${}^{\circ }$C. (**c**) Temperature changes in PAHVs (*L* = 5 mm) of different diameters (12–18 mm) when actuated at 100 ${}^{\circ }$C. (**d**) Temperature changes in PAHVs (*D* = 12 mm) of different lengths (3–7 mm) when actuated at 100 ${}^{\circ }$C. (**e**) PAHV temperature changes (*D* = 12 mm, *L* = 5 mm) when actuated at different temperatures (40–100 ${}^{\circ }$C).

The PAHV thermal profile was monitored by heating at 40–100 ${}^{\circ }$C for 30 min using a hot plate. The valves’ different outer diameters (*D* = 12–18 mm) and different thicknesses (*L* = 3–7 mm) were tested to observe their correlation. The PAHV surface was placed on the hot plate and the top surface’s temperature change was monitored using an IR imaging system. IR images as depicted in Figure 5a exhibit the valve’s top surface variation when the bottom surface was exposed to 100 ${}^{\circ }$C for 30 min. The valve’s top surface temperature was uniformly raised during the entire actuation process. Figure 5b demonstrated the PAHV’s heat transfer during the actuation process, estimated using FE simulations. Figure 5c shows that PAHVs with different outer diameters (*D* = 12–18 mm) reached the actuation temperature (100 ${}^{\circ }$C) at approximately the same time (30 min), demonstrating that the PAHV’s thermal actuation is independent of its outer diameter. The top surface temperature increased to ≈80 ${}^{\circ }$C within the first 15 min of the actuation, and the heating rate reduced during the next 15 min. PAHVs with different thicknesses displayed different heating rates when actuated at the same temperature (Figure 5d). The reduction in Valve thickness increased the PAHV’s heat transfer rate. The thicker PAHV (*L* = 7 mm) reached ≈90 ${}^{\circ }$C in 30 min when actuated at 100 ${}^{\circ }$C, whereas the thinner PAHV (*L* = 5 mm) reached ≈97 ${}^{\circ }$C in the same time, demonstrating faster actuation modalities. Decreasing the valve’s thickness further (*L* = 3 mm) lead to a negligible increase in the temperature (≈98 ${}^{\circ }$C), portraying an independent actuation rate with the valve’s thickness for *L* < 5 mm. Hence, PAHV with *L* = 5 mm tends to be the best thickness for the optimal actuation process. Figure 5e exhibits the valve’s top surface temperature change when actuated at different temperatures (40, 60, 80, and 100 ${}^{\circ }$C). The PAHV’s top surface reached the actuation temperature (40–100 ${}^{\circ }$C) within 30 min, demonstrating the tested actuation time to be sufficient for all actuation temperatures.

### 3.3. PAHV Thermo-Mechanical Performance

**Figure 6:**
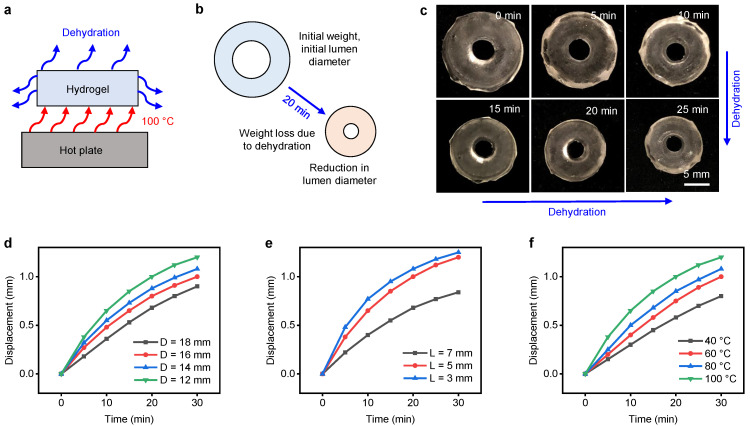
PAHV thermo-mechanical performance. Schematic showing the PAHV’s (**a**) experimental set-up, (**b**) de-swelling actuation mechanism. (**c**) Time-lapse images exhibiting the PAHV’s actuation dynamics (*D* = 12 mm, *L* = 5 mm) when heated at 100 ${}^{\circ }$C. (**d**) Displacement in PAHVs (*L* = 5 mm) of different diameters (12–18 mm) when actuated at 100 ${}^{\circ }$C. (**e**) PAHV displacement (*D* = 12 mm) for different lengths (3–7 mm) when actuated at 100 ${}^{\circ }$C. (**f**) PAHV displacement (*D* = 12 mm, *L* = 5 mm) when actuated at different temperatures (40–100 ${}^{\circ }$C).

We characterize PAHV performance by actuating it at 40–100 ${}^{\circ }$C for 30 min using a hot plate (Figure 6a). During thermal actuation, the PAHV dehydrates, resulting in deformation causing an inner lumen diameter change, resulting in a valve-like mechanism (Figure 6b). The PAHV’s actuation at 100 ${}^{\circ }$C for 30 min reduced the inner lumen diameter from 5 mm to ≈3.8 mm. Figure 6c exhibits the PAHV volume shrinkage when actuated at 100 ${}^{\circ }$C for 30 min. The PAHV’s inner lumen area change for different outer diameters (*D* = 12–18 mm) is calculated as displacement and shown in Figure 6d. The PAHV with *D* = 12 mm demonstrated the highest displacement (≈1.2 mm), while the PAHV with *D* = 18 mm showed the lowest displacement (≈0.9 mm), demonstrating the performance benefits from reduction of the valve’s outer diameter. The changes in PAHV performance for different thicknesses (*L* = 3–7 mm) are shown in Figure 6e. When actuated for 30 min, the PAHV with *L* = 3 mm and *L* = 5 mm showed a displacement of 1.25 mm and 1.2 mm, respectively, whereas the PAHV with *L* = 7 mm showed a displacement of only 0.84 mm. Although this demonstrates performance improvement by reducing the valve’s thickness, displacement increase rate reduced and started to saturate after *L* = 3 mm. Figure 6f exhibits the valve’s displacement when actuated at different temperatures (40, 60, 80, and 100 ${}^{\circ }$C) for 30 min. Although the PAHV’s temperature increased rapidly from 20 to 70 ${}^{\circ }$C in less than 10 min (Figure 5a–e), the PAHV’s volume change was minimal (Figure 6c) and the displacement was less than 0.5 mm (Figure 6d–f). The area rate limiting factor is the dehydration of the water content in the valve, which is further discussed in the forthcoming sections.

### 3.4. PAHV Dehydration Kinetics

**Figure 7:**
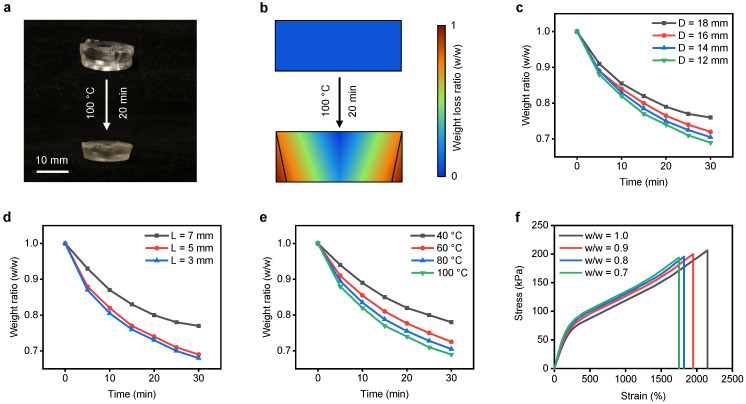
PAHV dehydration kinetics. (**a**) Real-time photos demonstrating PAHV’s dehydration kinetics (*D* = 12 mm, *L* = 5 mm) when heated at 100 ${}^{\circ }$C for 20 min. (**b**) Schematic showing the dehydration profile of the PAHV during actuation. (**c**) PAHV weight ratio change (*L* = 5 mm) for different diameters (12–18 mm) when actuated at 100 ${}^{\circ }$C. (**d**) PAHV weight ratio change (*D* = 12 mm) for different lengths (3–7 mm) when actuated at 100 ${}^{\circ }$C. (**e**) PAHV weight ratio change (*D* = 12 mm, *L* = 5 mm) when actuated at different temperatures (40–100 ${}^{\circ }$C). (**f**) PAHV extensometry performance at different weight ratios (w/w = 0.7, 0.8, 0.9, and 1.0).

During the PAHV’s thermal actuation, in addition to the inner lumen diameter change, the valve’s dehydration and weight loss were observed (Figure 7a). During actuation, the PAHV’s bottom surface is at a higher temperature than the top surface, causing nonuniform evaporation and weight loss (Figure 7a). The 2D shape changes on a temporal scale were quantified during the thermal actuation, and a profile showing the weight loss ratio was generated and is represented schematically in Figure 7b. A precision balance (EP 125SM, Precisa Gravimetrics, Dietikon, Switzerland) was used to determine the PAHV’s weight loss during the actuation process. PAHV actuation at 100 ${}^{\circ }$C for 30 min showed the PAHV’s ≈20–30% weight loss when *D* = 18–12 mm (Figure 7c). The influence of valve height on dehydration and weight loss during actuation was tested with *L* = 3–5 mm and shown in Figure 7d. The influence of different actuation temperature (40, 60, 80, and 100 ${}^{\circ }$C) for weight loss during actuation for 30 min was also tested, and the results are shown in Figure 7e. To examine the influence of dehydration on the PAHV’s mechanical properties, we conducted uniaxial testing using a universal testing machine (5543, Instron) with a 500 N load cell at a rate of 5 mm min${}^{-1}$. A fresh PAHV (w/w = 1.0) achieved a maximum strain of ≈2150% before fracture, sustaining a stress of 206 kPa (Figure 7f), whereas PAHVs with different dehydration degrees (10–30%) demonstrate reduced elasticity. PAHVs with 10% dehydration (w/w = 0.9), 20% dehydration (w/w = 0.8), and 30% dehydration (w/w = 0.7) achieved maximum strain of ≈1950%,≈1820%, and ≈1750%, sustaining stresses of 200 kPa, 196 kPa, and 194 kPa, respectively (Figure 7f).

### 3.5. PAHV Rehydration Kinetics

**Figure 8:**
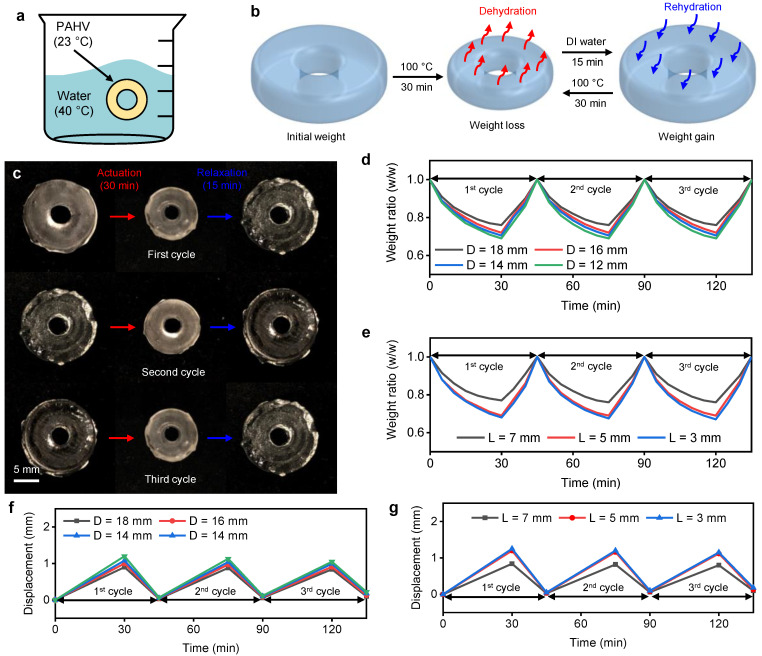
PAHV rehydration/controlled swelling kinetics. (**a**) Schematic showing the experimental set-up and (**b**) actuation and rehydration mechanisms. (**c**) Real-time images demonstrating actuation and rehydration of the PAHV’s actuation and rehydration cycles. (**d**) PAHV weight ratio changes for different diameters (12–18 mm) actuated at 100 ${}^{\circ }$C and rehydrated in DI water for 3 cycles. (**e**) PAHV weight ratio changes for different lengths (3–7 mm) actuated at 100 ${}^{\circ }$C and rehydrated in DI water for 3 cycles. (**f**) PAHV displacement at different diameters (12–18 mm) for 3 actuation–rehydration cycles. (**g**) PAHV displacement at different thicknesses (3–7 mm) for 3 actuation–rehydration cycles.

Although the PAHV’s weight loss is ≈20–30% during 30 min thermal actuation (100 ${}^{\circ }$C), the hydrogel’s swelling nature allows the valve to revert to its initial structure by immersing it in DI water for 15 min (Figure 8a). The PAHV’s volumetric change during 30 min actuation and 15 min rehydration is shown schematically in Figure 8b. To test the method’s reproducibility, we actuated the PAHV for 30 min followed by rehydration for 15 min for three cycles. Due to the valve’s nonuniform dehydration behavior, the PAHV’s small structural changes were observed over the three actuation–rehydration cycles (Figure 8c). Despite these structural changes, the PAHV’s weight loss during 30 min thermal actuation and the weight gain during 15 min rehydration in DI water remained approximately the same during all three cycles (Figure 8d–e). This trend is observed with different diameters (*D* = 12–18 mm, Figure 8d) and different thicknesses (*L* = 3–7 mm, Figure 8e). We further tested PAHV’s performance for three actuation–rehydration cycles to determine the consistency in its displacement. Figure 8f–g shows ≈1–10% loss in displacement over three cycles for PAHV with different diameters (*D* = 12–18 mm, Figure 8d) and different thicknesses (*L* = 3–7 mm).

## 4. NPHV and PAHV Performance Comparison

**Figure 9:**
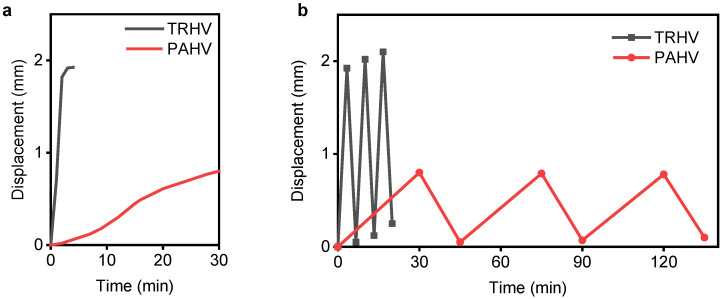
NPHV and PAHV thermo-mechanical performance comparison. (**a**) The NPHV’s and PAHV’s displacement during actuation at 40 ${}^{\circ }$C. (**b**) The NPHV’s and PAHV’s displacement for three actuation cycles.

Here, we summarize and compare the NPHV’s and PAHV’s annular actuation performance as also depicted in Figure 4 and Figure 6. The NPHV (*D* = 12 mm, *L* = 5 mm) and PAHV (*D* = 12 mm, *L* = 3 mm) were actuated at 40 ${}^{\circ }$C and their displacement performance was observed. The NPHV can achieve a displacement of ≈1.93 mm in 4 min, whereas PAHV exhibited a displacement of 0.8 mm in 30 min (Figure 9a). Both the NPHV and PAHV emulated consistent displacement performance for three actuation cycles when actuated for 20 min and 135 min, respectively (Figure 9b). Although the NPHV outperformed PAHV in displacement, when actuated at different temperatures, the PAHV can be used for applications including fluid flow control and gripping, as we further discuss in the forthcoming section. A force transducer (Nano17-E, ATI Industrial Automation, Apex, NC, USA) was used to determine the force exerted by the PAHV during the thermal actuation process. The PAHV exerts a force of ≈0.1–0.2 N when actuated at 40–100 ${}^{\circ }$C, which can be used for the fluid flow control and gripping applications.

## 5. Applications

### 5.1. PAHV Fluid Flow Control Valve

**Figure 10:**
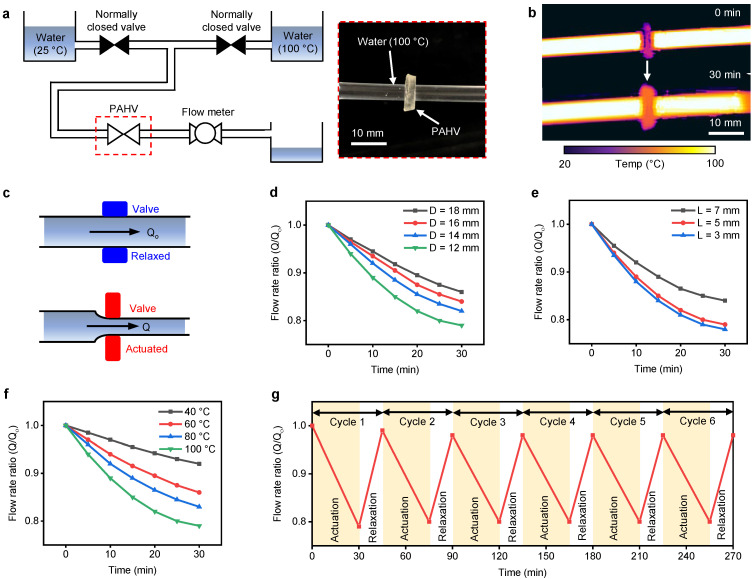
PAHV performance for flow control application. (**a**) Real-time photograph showing the PAHV’s flow control system setup. (**b**) IR images showing the actuation of the PAHV flow control valve when the fluid is at 100 ${}^{\circ }$C. (**c**) Schematic demonstrating the PAHV’s flow control valve mechanism. (**d**) PAHV flow rate (*L* = 5 mm) for different diameters (12–18 mm) when fluid is at 100 ${}^{\circ }$C. (**e**) PAHV flow rate (*D* = 12 mm) for different lengths (3–7 mm) when fluid is at 100 ${}^{\circ }$C. (**f**) PAHV flow rate (*D* = 12 mm, *L* = 5 mm) when fluid is at different temperatures (40–100 ${}^{\circ }$C). (**g**) Flow rate in PAHV (*D* = 12 mm, *L* = 5 mm) for 6 cycles when actuating fluid is at 100 ${}^{\circ }$C.

To demonstrate the PAHV’s capability to control fluid flow, we designed a simple flow control system, as shown in Figure 10a. Two reservoirs of water, one at room temperature (25 ${}^{\circ }$C) and another heated to 100 ${}^{\circ }$C using a hot plate were used as the PAHV’s functional fluid. Two normally-closed valves were used to control the fluid flow between the two reservoirs. A flow meter was attached at the outlet of the PAHV to measure the flow rate. All the components in the flow control system were connected using a flexible tube (*OD* = 4 mm, *ID* = 3 mm). When hot water (100 ${}^{\circ }$C) passed through the flexible tube, heat from the water was transferred to the PAHV, thereby actuating the valve (Figure 10b). This actuation lead to displacement in the PAHV, thereby contracting the flexible tube, resulting in the reduction of fluid flow (Figure 10c). Initially, we allowed water at room temperature (25 ${}^{\circ }$C) to pass through PAHV and we measured the flow rate (*Q${}_{0}$*) at its outlet using the flow meter. We then stopped the water (room temperature) flow and allowed the hot water (100 ${}^{\circ }$C) to pass through the PAHV, and recorded the flow rate (*Q*) every 5 min. By monitoring the system’s fluid flow rate during actuation (*Q*) and un-actuation states (*Q${}_{0}$*) of the PAHV, we calculated the flow rate ratio (*Q/Q${}_{0}$*). After actuation for 30 min, PAHVs of different diameters (*D* = 12–18 mm) showed a variation in *Q/Q${}_{0}$* of ≈10–20% (Figure 10d). Varying thicknesses of the PAHV (*L* = 3–7 mm) showed a change in *Q/Q${}_{0}$* of ≈12–20% (Figure 10e). Varying the fluid temperature from 100 ${}^{\circ }$C to 40 ${}^{\circ }$C displayed a difference in *Q/Q${}_{0}$* of ≈13% (Figure 10f).

To examine the reproducibility of the fluid flow control, we record the flow rate during maximum actuated state and completely relaxed state for six cycles. Initially, we allowed water at room temperature (25 ${}^{\circ }$C) to pass through the PAHV (*D* = 12 mm, *L* = 5 mm) and we measured the flow rate (*Q${}_{0}$*). We then stopped the water (room temperature) flow and allowed the hot water (100 ${}^{\circ }$C) to pass through the PAHV for 30 min and we recorded the flow rate ratio (*Q/Q${}_{0}$*). We then stopped the hot water flow and allowed the room temperature water to flow through the PAHV while immersing the PAHV in DI water for 15 min to allow rehydration, and we recorded the flow rate ratio. We performed this procedure for six cycles and observed that the variation in flow rate ratio remained consistent for each cycle (Figure 10g). Although the PAHV demonstrated the ability to control fluid flow, its slow response time limits its potential in applications requiring high-frequency actuation.

### 5.2. PAHV Circumferential Gripper

**Figure 11:**
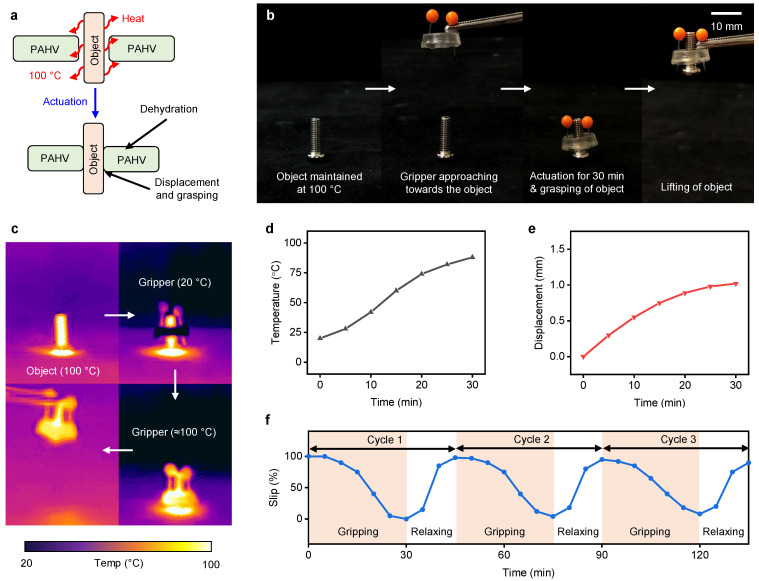
PAHV performance in a gripping application. (**a**) Schematic showing grasping mechanism of the PAHV gripper. (**b**) Time-lapse images demonstrating the PAHV grasping. (**c**) IR images exhibiting the PAHV gripper’s actuation when the object is at 100 ${}^{\circ }$C. (**d**) Temperature in the PAHV when the object is at 100 ${}^{\circ }$C. (**e**) Displacement in the PAHV when the object is at 100 ${}^{\circ }$C. (**f**) Object slipping in the PAHV gripper during grasping for 3 cycles when object is at 100 ${}^{\circ }$C.

To demonstrate the PAHV’s capability to grasp small objects (≈1 g), the PAHV (*D* = 12 mm, *L* = 3 mm) was used to grasp and lift a threaded bolt (≈1 g). The PAHV’s actuation and working mechanism as a circumferential gripper for grasping objects is shown in Figure 11a. The object (threaded bolt) is maintained at 100 ${}^{\circ }$C, and when the PAHV comes into contact with the object, heat is transferred to the PAHV, thereby actuating the valve. During 30 min actuation, the PAHV grasped the object, demonstrating its ability to lift the object. Sequential steps involved in the process of gripping and lifting a threaded bolt (1 g) using a PAHV are shown in Figure 11b. The PAHV’s temperature profile for a circumferential gripper during actuation was monitored using an IR imaging system and the entire actuation process was video-recorded. IR images showing heat transfer from the object to the PAHV and corresponding contraction, leading to gripping and lifting, are displayed in Figure 11c. When the PAHV was in contact with the object (100 ${}^{\circ }$C) for 30 min, the PAHV circumferential gripper reached ≈88 ${}^{\circ }$C (Figure 11d) and demonstrated a displacement of ≈1 mm (Figure 11e).

To examine the reproducibility of the gripping action in the PAHV circumferential gripper, we performed three gripping–relaxing cycles. Initially, we brought the PAHV into contact with the object (100 ${}^{\circ }$C), and then lifted the gripper and measured the percent of slipping in the object every 5 min. After 30 min of actuation, the PAHV was immersed in DI water for 15 min, with slipping measured every 5 min. We performed this procedure for three cycles and observed the variation in the object’s slipping. During the gripping/actuation cycle, the object slipped completely during the first 5 min, followed by a slow decrease in slipping, reaching ≈0% slipping at 25 min (Figure 11f). During the relaxation cycle, the first 5 min exhibited a small increase in slipping, followed by a drastic increase in slipping to ≈90% in the next 5 min, followed by a slow increase and saturation at ≈100% in the next 5 min.

## 6. Conclusions

In summary, herein, we present a performance comparison of the NPHV and PAHV concerning their applications. When heated at ≈40 ${}^{\circ }$C, the NPHV performs at its best with ≈1.93 mm displacement in 4 min, whereas the PAHV exhibits 0.8 mm in 30 min. Although the NPHV outperforms the PAHV in displacement, the PAHV demonstrates the ability to be used for applications such as fluid flow control and circumferential gripping. The PAHV also demonstrates fast rehydration capability by reverting to the original weight in just 15 min (50% of actuation time). The PAHV flow control valve demonstrates a flow rate change of up to 20% for six cycles when the input fluid is at 100 ${}^{\circ }$C. The PAHV gripper (≈1 g) demonstrates the ability to grip a threaded bolt (≈1 g) and lift it securely within 30 s when the object is at 100 ${}^{\circ }$C. This work will pave the way for a better soft, flexible, highly robust biocompatible valve system that can be used for diversified biomedical applications, especially in minimally-invasive surgeries, controlled drug delivery, soft biomedical grippers, and tissue ablation. 

## Data Availability

Data available on request.

## Abbreviations

The following abbreviations are used in this manuscript:

NPHV	N-Isopropylacrylamide hydrogels-based valve
PAHV	PAAm-alinate hydrogels-based valve
